# Receptors and Channels Possibly Mediating the Effects of Phytocannabinoids on Seizures and Epilepsy

**DOI:** 10.3390/ph13080174

**Published:** 2020-07-30

**Authors:** Lara Senn, Giuseppe Cannazza, Giuseppe Biagini

**Affiliations:** 1 Laboratory of Experimental Epileptology, Department of Biomedical, Metabolic and Neural Sciences, University of Modena and Reggio Emilia, 41125 Modena, Italy; lara.senn@studenti.unimore.it; 2 Department of Life Sciences, University of Modena and Reggio Emilia, 41125 Modena, Italy; giuseppe.cannazza@unimore.it; 3 Center for Neuroscience and Neurotechnology, University of Modena and Reggio Emilia, 41125 Modena, Italy

**Keywords:** phytocannabinoids, epilepsy, anticonvulsant, cannabis, seizure

## Abstract

Epilepsy contributes to approximately 1% of the global disease burden. By affecting especially young children as well as older persons of all social and racial variety, epilepsy is a present disorder worldwide. Currently, only 65% of epileptic patients can be successfully treated with antiepileptic drugs. For this reason, alternative medicine receives more attention. Cannabis has been cultivated for over 6000 years to treat pain and insomnia and used since the 19th century to suppress epileptic seizures. The two best described phytocannabinoids, (−)-*trans*-Δ^9^-tetrahydrocannabinol (THC) and cannabidiol (CBD) are claimed to have positive effects on different neurological as well as neurodegenerative diseases, including epilepsy. There are different cannabinoids which act through different types of receptors and channels, including the cannabinoid receptor 1 and 2 (CB_1_, CB_2_), G protein-coupled receptor 55 (GPR55) and 18 (GPR18), opioid receptor µ and δ, transient receptor potential vanilloid type 1 (TRPV1) and 2 (TRPV2), type A γ-aminobutyric acid receptor (GABA_A_R) and voltage-gated sodium channels (VGSC). The mechanisms and importance of the interaction between phytocannabinoids and their different sites of action regarding epileptic seizures and their clinical value are described in this review.

## 1. Introduction

Epilepsy is a chronic neurological disease affecting approximately 50 million people of all ages and sexes worldwide. This spectrum disorder not only impairs neuronal circuits, but also leads to social burden and severe morbidity showing highest incidence in young children and the elderly [1]. Epileptic seizures are defined as paroxysmal electrical discharges originated from various brain regions, leading to molecular, physiological, cognitive and social dysfunction [2]. The origin is thought to lie in the imbalance of the activation of excitatory and inhibitory synapses due to several causes, including genetic disorders, stroke, infections, injuries etc. According to the International League Against Epilepsy (ILAE), seizures can be classified in two groups: focal (or partial) and generalized. Focal seizures initiate in small groups of neurons in one hemisphere of the brain, or of one lobe, resulting in jerks and clonic movements. Generalized seizures involve both hemispheres from the onset and might lead to tonic-clonic movements and loss of consciousness and posture. Furthermore, epilepsy can be split into primary and secondary epilepsy. Primary epilepsy is of unknown cause without any previous physiological or molecular damage of the brain, whereas secondary epilepsy might occur as a result of neurological etiologies including those structural, genetic, infectious, metabolic, or immune [3,4].

Epileptic seizures can be caused by an imbalance of inhibitory and excitatory activity as shown by drugs able to block inhibitory type A y-aminobutyric acid receptor (GABA_A_R) and the corresponding synaptic currents [5], or by activating ligand-gated and voltage-gated excitatory synaptic currents [6,7]. Thus, seizures might be limited by an increase of inhibitory currents [8] or decrease of excitatory currents [9,10]. However, in recent years many researchers have investigated this disease, but the exact mechanisms and ultimate cure still remains to be elucidated.

With the notable exception of absence seizures, anticonvulsants used for different epilepsy types act by enhancing inhibitory drive or by counteracting excitatory activity. Antiepileptic drugs (AEDs) have been used as a successful treatment for approximately 65% of suffering patients [11]. For instance, lorazepam and other benzodiazepines increase GABA_A_R-mediated inhibition and thus display a decent medication for symptomatic seizures [12,13]. Other medications such as phenytoin and carbamazepine prevent voltage-gated Na^+^ channels from activation and therefore reduce the firing of action potentials [14,15]. Nevertheless, these first-line medications are linked to strong-side effects and tolerability. In 1886, the first surgical approach for epilepsy was done by Horsley Victor, which expanded the therapeutic possibilities for epileptic seizures. He resected cortical tissue adjacent to a depressed skull fracture and healed a patient suffering from focal motor seizures [16]. Nowadays, for 35% of drug-resistant patients with refractory epilepsy, invasive treatments including surgical resection or neurostimulation have been demonstrated to be the only chance for cure. Thus, invasive treatments often appear as the ultimate prospect for these patients.

In the last years, the need for therapies for refractory seizures has largely arisen, which lead researchers to expand their mind to investigate more in alternative eligible treatments. In recent studies phytocannabinoids have been tested as an alternative approach for patients suffering from refractory seizures.

## 2. *Cannabis sativa* L. and Its Major Derivatives

*Cannabis sativa* L. has shown its medical potential for more than 6000 years, during which it spread from Northwestern Asia to Europe and finally to all over the world. The first historical evidence of medicinal use of *C. sativa* was recorded in the herbal medicine *Pên-ts’ao Ching* described by the Emperor Shen Nung around 2000 BC [17]. Furthermore, in ancient Greece and Rome the plant was used to treat pain, spasm and cramps [18]. The genus *Cannabis* belongs to the family of Cannabaceae. The taxonomy proposed by Small and Cronquist combining morphological and chemical description, considered Cannabis as monospecific (*Cannabis sativa* L.) with two subspecies (*Cannabis sativa* L. subsp. *sativa*, and *Cannabis sativa* L. subsp. *indica*) and four varieties (*Cannabis sativa* L. subsp. *sativa* var. *sativa*; *Cannabis sativa* L. subsp. *sativa* var. *spontanea*; *Cannabis sativa* L. subsp. *indica* var. *indica*; *Cannabis sativa* L. subsp. *indica* var. *kafiristanica*) [19]. Cannabis contains a characteristic class of isoprenylated resorcinyl polyketides compounds called phytocannabinoids to distinguish them from synthetic and endogenous cannabinoids. Notwithstanding phytocannabinoids are more characteristic of Cannabis, there are reports in the literature that phytocannabinoids also occur in other plants such as Helichrysum [20]. One hundred and fifty phytocannabinoids have been recorded for *C. sativa* to date and can be classified into 11 general types: (−)-*trans*-Δ^9^-tetrahydrocannabinol (THC), (−)-*trans*-Δ^8^-tetrahydrocannabinol (Δ^8^-THC), cannabigerol (CBG), cannabichromene (CBC), cannabidiol (CBD), cannabinodiol (CBND), cannabielsoin (CBE), cannabicyclol (CBL), cannabinol (CBN), cannabitriol (CBT), and miscellaneous types [20]. THC and CBD are the most important and studied plant cannabinoids. In 1940, CBD was first isolated from the plant [21]. In 1963 its structure was first described [22] and followed by its first identification as a crystal structure in 1977 [23]. The major breakthrough in cannabinoid research was achieved by Mechoulam and Gaoni in 1964 with the identification of the chemical structure of the first described psychoactive phytocannabinoid THC [24]. Cannabis varieties can be classified into five different chemotypes depending on the concentration of the main phytocannabinnoids. Drug-type cannabis varieties that have a high THC/CBD ratio (≫1.0) are classified as chemotype I; varieties with an intermediate ratio (0.5–2.0) are classified as chemotype II; fiber-type varieties that have a low THC/CBD ratio (≪1.0) are classified as chemotype III; chemotype IV are cannabis varieties that contain CBG as the main cannabinoid; and chemotype V cannabis fiber-type varieties that contain almost no cannabinoids [25].

**Figure 1:**
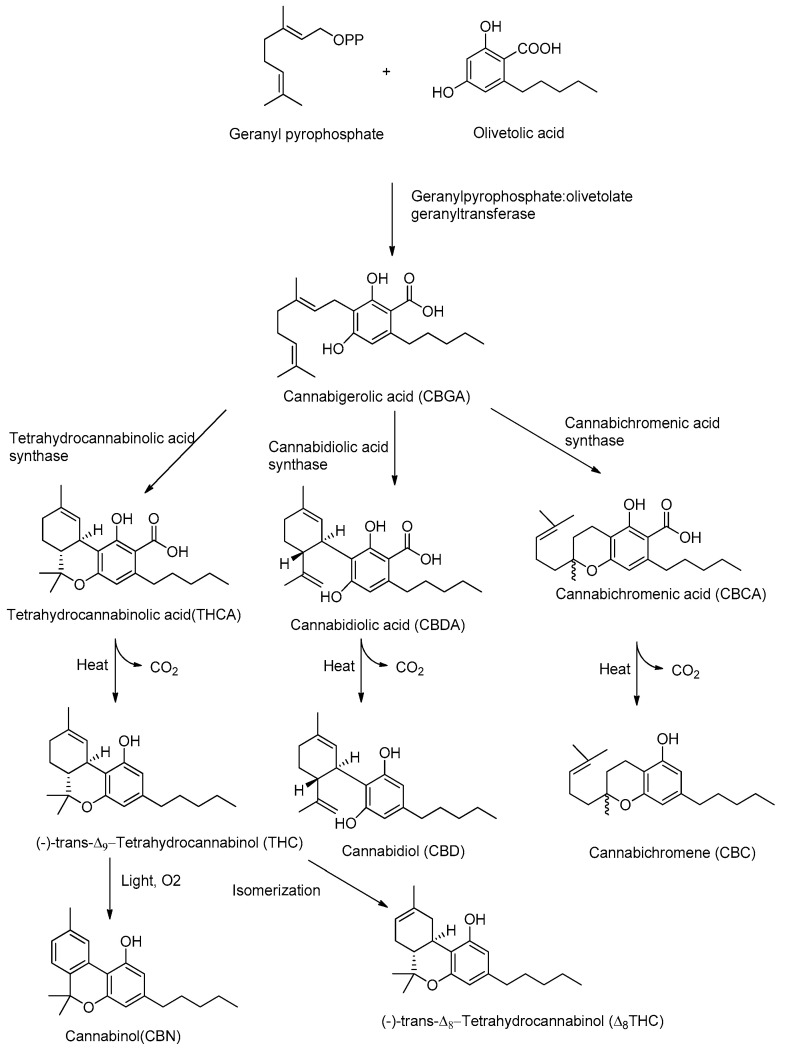
Biosynthetic pathway of major phytocannabinoids.

Although CBD and THC have long been considered authentic natural products of cannabis, these molecules are not enzymatically synthesized in the plant, which instead produces cannabidiolic acid (CBDA) and tetrahydrocannabinolic acid (THCA) (Figure 1).

Today it is accepted that CBD and THC are an unnatural artifact of the corresponding acid precursors CBDA and THCA, produced via a temperature-catalyzed reaction. The different phytocannabinoids in plants originate from a common precursor, which is cannabigerolic acid (CBGA), in turn obtained by the alkylation of olivetolic acid with geranyl pyrophosphate (Figure 1) [26,27]. The other phytocannabinoids are biosynthesized from CBGA by the action of oxidoreductase enzymes, such as THCA-synthase, CBDA-synthase and cannabicromenic acid (CBCA)-synthase, which lead to the formation of phytocannabinoids such as THCA, CBDA and CBCA [28]. These carboxylated cannabinoids, so-called “acids”, are easily decarboxylated into the corresponding “neutral” derivatives, such as THC, CBD and CBC, with a non-enzymatic reaction of decarboxylation catalyzed by heat [29]. There are also different phytocannabinoids resulting from oxidation or isomerization of THC and CBD, such as CBN or Δ^8^-THC.

THC is the main phytocannabinoid of cannabis which is responsible for the psychoactive properties such as psychotropic effects, including euphoria, appetite enhancement and alteration of sensory perception. Since the chemical structure of THC was elucidated by Mechoulam in 1964, a large number of scientific papers have been published concerning its mechanism of action. Furthermore, its structure was taken as a lead compound for the development of increasingly active synthetic cannabinoids. Such synthetic cannabinoids have different chemical structures like classical (e.g., nabilones) [30], non-classical (e.g., WIN55212-2) [31], aminoalkylindoles (e.g., JWH-018) [32] and endogenous arachidonic acid derivatives including endocannabinoids such as 2-arachidonoylglycerol (2-AG) and N-arachidonylethanolamide (AEA [33,34,35]. THC does not only exhibit positive effects in the treatment of severe pain and nausea [36] but was also shown to inhibit T-cell immune-response, as well as to diminish inflammatory cytokine and chemokine release in rat microglia [37]. However, the use of cannabis is associated with abuse potential leading to behavioral changes and psychological impairment, because of the pharmacological properties of THC [38,39]. Therefore, THC is still not considered as a reliable, predictable and safe long-term derivative to treat neurological diseases such as epilepsy or depression [40]. Alternatively, to THC, CBD represents a promising tool against refractory epilepsy as it lacks the psychoactive properties and presents few side effects. A reduced occurrence of seizure discharges in rats has been demonstrated in the hippocampus treated with CBD [41]. It has been shown that CBD exerts beneficial effects to restore the activity of hippocampal neurons [42] and prevents neuronal cell death in temporal lobe epilepsy (TLE) models [43]. It is furthermore observed that CBD, as THC, shows high anti-inflammatory [44], antioxidant, and anticonvulsant activity [45,46]. The cannabis-derived product Epidiolex^®^ (GW Pharmaceuticals, Cambridge, UK) was approved in 2018 by the US Food and Drug Administration (FDA) for the treatment of the rare pediatric onset refractory epilepsy disorders Dravet syndrome (DS) and Lennox-Gastaut syndrome (LGS) [47]. Currently, Epidiolex^®^, which is composed of 100 mg/mL of CBD in sesame oil, has been tested in clinical trials with children, obtaining a 36.5% median reduction in monthly motor seizures during a 12-week treatment period. Adjunctive CBD could even raise the number to approximately 50% of seizure reduction during a total of 96-weeks. Even though this open-labeled study observed very few side effects (5%) and promising outcomes, one should be reminiscent about the placebo effect of this medication associated with parental expectation and media attention put on the children. Children, which moved across the US country to receive the treatment were twice as likely to decrease seizure events/month compared to children who happened to be living in the area of the medical center (47% versus 22%) [48,49].

**Figure 2:**
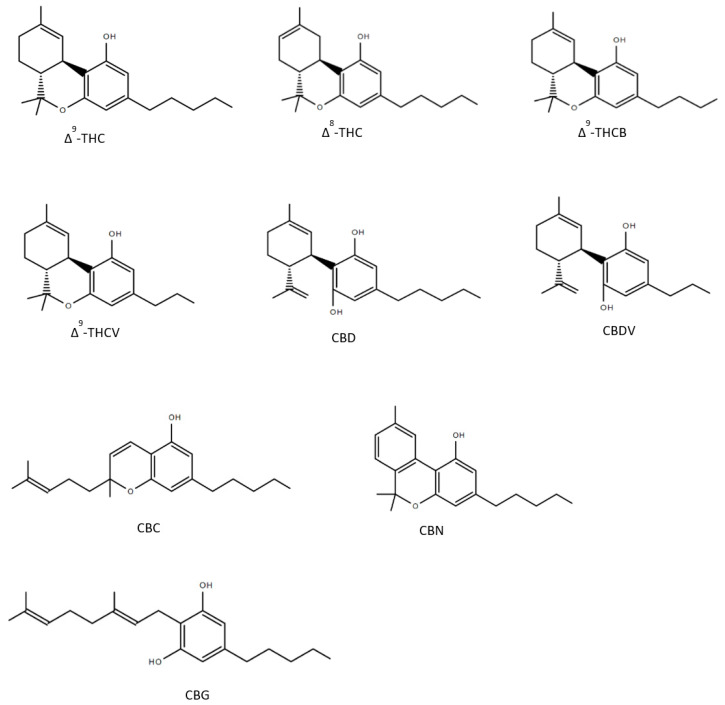
Structures of nine phytocannabinoids showing anticonvulsant activity; Δ^9^-tetrahydrocannabinol (Δ^9^-THC), Δ^8^-tetrahydrocannabinol (Δ^8^-THC), Δ^9^-tetrahydrocannabutol (Δ^9^-THCB), Δ^9^-tetrahydrocannabivarin (THCV), cannabidiol (CBD), cannabidivarin (CBDV), cannabichromene (CBC), cannabinol (CBN), cannabigerol (CBG).

Until now over 150 compounds of *Cannabis sativa* could be identified as phytocannabinoids of which a few numbers have already been tested to reveal healing properties (Figure 2). Δ^9^-tetrahydrocannabutol (Δ^9^-THCB), which is the butyl homologue of THC showed possible anti-inflammatory and analgesic activity in a model of acute inflammatory pain [50]. As THC, Δ^8^-THC presents psychoactive effects and in the first experiments promisingly diminished the growth of lung adenocarcinoma both in vitro and in vivo [51,52]. The propyl analogue of THC, Δ^9^-tetrahydrocannabivarin (THCV) is an antioxidant [53] and shows symptom-relieving and neuroprotective effects in animal models of Parkinson’s disease [54]. As well as the propyl analogue of CBD named cannabidivarin (CBDV) offers medical advantages: CBDV is able to rescue motor impairment, cognitive dysfunction and brain atrophy in a mouse model of Rett syndrome [55]. Further compounds of the cannabis plant, which count to the most abundant cannabinoids are CBG and CBC: several studies observed that both CBG and CBC could be used against neuroinflammation, oxidative stress and exhibit analgesic effects [56,57,58,59,60]. As CBD, CBN has been detected to have many valuable responses against inflammation [61], convulsions [62] and pain [63]. The structures of the phytocannabinoids are presented in Figure 2.

There are still many cannabinoids which have been isolated and described in the last years, but still remain to be experimentally explored.

## 3. A Brief Summary of the Endocannabinoid System (ECS)

The ECS is a key modulatory system involving the cannabinoid receptor 1 (CB_1_) and 2 (CB_2_), their endogenous ligands and the enzymes responsible for their biosynthesis and inactivation. It has been suggested that the ECS plays an important role in the neuroprotection of acute neurological diseases, such as epilepsy, as well as chronic neurodegenerative diseases such as Parkinson’s disease [64,65].

The discovery of CB_1_ and CB_2_ receptors in the central nervous system (CNS) opened the field for the exploration of endogenous regulating systems and compounds associated with physiological processes and neurological disorders involving the endocannabinoids. CB_1_ is mostly sited on presynaptic inputs in several parts of the brain, including the olfactory bulb, the cerebral cortex and corpus striatum, and is highly expressed also in the hippocampus. In the dentate gyrus and CA3 hippocampal subfield, especially in the stratum oriens, highly dense receptor binding sites have been observed [66]. Conversely, CB_2_ receptors are mainly expressed in cells of the immune and hematopoietic system, but they have been previously discovered in neurons of the brain stem [67]. In 1992, the first endogenous ligand of CB_1_ was described and named anandamide (i.e., AEA) [34]. Three years later, 2-AG also was identified [33]; both are described as the most abundant endogenous ligands for CB_1_ and CB_2_. Anandamide primarily targets CB_1_, while 2-AG shows agonistic effects on both CB_1_ and CB_2_ receptors [68]. Anandamide and 2-AG are important mediators of synaptic plasticity and are synthesized by the lipid precursors N-arachidonoyl phosphatidylethanolamine (NAPE) and diacylglycerol (DAG) in the cell membrane [69]. They are released “on demand” by physiological or pathological stimuli and act as retrograde messengers [70]. Their signaling pathway also may be initiated by the depolarization of a postsynaptic neuron, which opens voltage-gated calcium channels (VDCCs) leading to increased cytoplasmic calcium so to trigger endocannabinoid synthesis and release from the postsynaptic cell by a yet unknown mechanism. Endocannabinoids diffuse retrogradely to a presynaptic bouton and bind to receptors reducing the likelihood of release of the excitatory and inhibitory neurotransmitters [71,72]. Anandamide is hydrolyzed to arachidonic acid (AA) and ethanolamine by fatty acid amide hydrolase (FAAH), while 2-AG is hydrolyzed to AA and glycerol by monoacylglycerol lipase [73,74]. Both these endocannabinoids have been found to play a significant role in the regulation of excitatory synapses suggesting the impairment of endocannabinoid signaling being linked to epilepsy. It has been described that CB_1_ and diacylglycerol lipase α are downregulated in epileptic human hippocampi [75]. The extracellular accumulation of 2-AG or anandamide was related to an anticonvulsant effect in the rat model of pentylenetetrazole-induced tonic-clonic seizures [76]. Additionally, in patients suffering from TLE lower concentrations of anandamide were found in the cerebrospinal fluid [77]. Therefore, inhibition of the breakdown of 2-AG and especially anandamide has been investigated lately as a new pharmaceutical target against epileptic seizures. The FAAH inhibitor URB597 was able to prevent or diminish alterations evoked by seizures in a kainic acid mouse model of TLE [78]. According to a study on cocaine-induced seizures in mice, URB597 inhibited seizure activity and showed a neuroprotective activity against seizure-related cell death [79]. However, the mechanism and function of inhibition of endocannabinoid hydrolysis in epileptogenesis requires further investigation.

## 4. Anticonvulsant Effects of Phytocannabinoids on Diverse Targets

**Figure 3:**
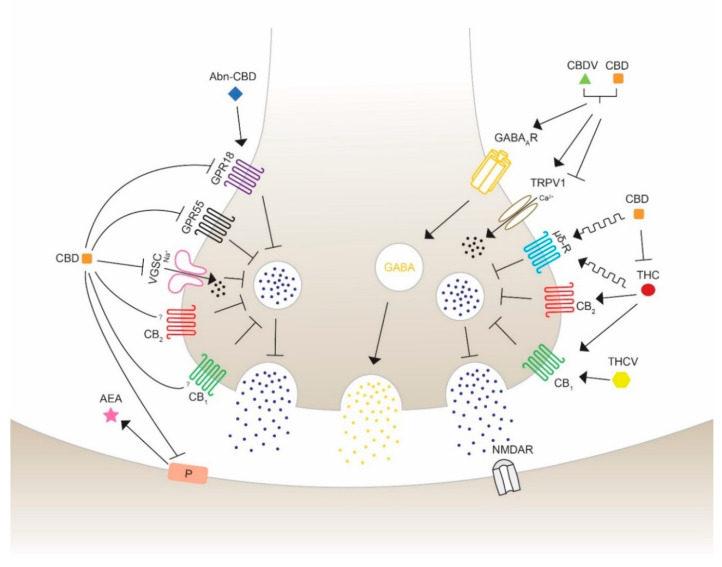
Schematic overview of the action of different phytocannabinoids possibly able to modulate seizures and epilepsy. Cannabidiol (CBD) inhibits the synthesis and mobilization of *N*-arachidonoylethanolamide (AEA) from the postsynaptic synapses, therefore acting in an independent and indirect antagonist manner with respect to the cannabinoid receptors CB_1_ and CB_2_. CBD antagonizes the activity of G protein-coupled receptor 18 (GPR18) and 55 (GPR55) and Na^+^ influx of voltage-gated sodium channels (VGSC) to block neurotransmission activity. Abnormal-CBD (Abn-CBD) acts through GPR18 to decrease intracellular Ca^2+^ release (left side). CBD and CBDV are proposed to show positive modulation and agonist effects on the type A γ-aminobutyric acid receptor (GABA_A_R), leading to an activation of GABA mobilization on inhibitory synapses. CBD and cannabidivarin (CBDV) activate and desensitize the transient receptor potential vanilloid type 1 and 2 (TRPV1/2), reducing extracellular Ca^2+^ influx and decreasing Ca^2+^ concentration. (−)-*trans*-Δ^9^-tetrahydrocannabinol (THC) and CBD are allosteric modulators of the opioid receptor type µ and δ, which inhibits the release of neurotransmitters to activate the glutamatergic *N*-methyl-d-aspartate (NMDA) receptor leading to a seizure reduction. THC activates CB_1_ and CB_2_ leading to an inhibition of glutamate release. This action can be blocked by CBD, which is able to inhibit THC-CB_1_ interaction. Δ^9^-tetrahydrocannabivarin (THCV) is suggested to induce anticonvulsant activity in a concentration- and CB_1_-mediated manner. The exact mechanisms of activation, inhibition or modulation are still under consideration. Furthermore, the different potency of the indicated molecules should be taken into account to correctly interpreter the illustrated effects, as in the case of THC and CBD which bind to CB receptors with affinity, respectively, in the nM and mM range.

The use of cannabis in neurological and neurodegenerative disorders is controversial and, thus, still under consideration. THC and CBD have shown in numerous preclinical studies to diminish epileptic seizures, thus increasing their medical interest. They are able to regulate the excitability of neuronal circuits involving the ECS and associated ligands and receptors. CBD has been proved to act as a reuptake inhibitor of anandamide, changing the excitatory and inhibitory dynamics of synapses [80]. The fact that phytocannabinoids not only exhibit agonist and antagonist actions leads to the understanding that the wide range of targets could reveal opposing and unpredictable effects. Therefore, the major exploration to specify the pharmacological targets of cannabinoids is crucial for the development of medicines for specific disorders. The promising beneficial health effects encourages many researchers in testing the possible therapeutic properties on seizures using phytocannabinoids with a chemical structure similar to THC and to CBD, such as Δ^8^-THC, Δ^9^-THCB, Δ^9^-THCV, CBDV, CBN. The anticonvulsant properties of cannabinoids acting through different receptors and channels are depicted and visualized (Figure 3) as follows.

### 4.1. G Protein-Coupled Receptors

#### 4.1.1. Cannabinoid Receptors CB_1_ & CB_2_

In 1991, the cannabinoid receptor CB_1_ was first described in the brain by receptor autoradiography and was identified as a G protein-coupled receptor [81,82]. The majority of CB_1_ receptors are located on presynaptic boutons of GABAergic interneurons [83,84], but can also be found in glutamatergic synapses [85]. Their main task is to reduce the likelihood of neurotransmitter release through various mechanisms, such as inhibition of calcium influx and adenylyl cyclase activity, or activation of the presynaptic potassium channels [86]. CB_1_ receptors are the most abundant receptors in the human and murine brain, including the olfactory bulb, hippocampus, amygdala, cerebellum, neocortex and basal ganglia, but are also found in peripheral tissues and cells [66,87]. Sequence analysis showed that CB_1_ sequence identity of humans and mice matches 97%, indicating the mouse as a reliable model for researching the CB_1_ receptor [88]. In 2005, CB_2_ receptors were first observed in the CNS, but their density is much lower than CB_1_ and they are mainly located on microglia and specific neurons [67,89,90]. CB_2_ receptors are primarily found in hematopoietic and immune cells including B-cells, T-cells and macrophages [91], having their major task in regulating the cytokine release [92]. CB_1_ and CB_2_ are the main targets of the endogenous ligands anandamide and 2-AG; whereas anandamide mainly binds CB_1_, 2-AG shows agonist effects on both receptors [68]. Interestingly, a small number of phytocannabinoids have demonstrated to possess seizure-diminishing effects acting through both CB_1_ and CB_2_ receptors.

CBD is suspected to act also by interacting with targets different from CB_1_ and CB_2_ receptors, suggesting the involvement of alternative transduction mechanisms [93,94,95]. Due to the inhibition of breakdown of anandamide, CBD may have an indirect mechanism to reveal its anticonvulsant activity [80]. Moreover, CBD has even shown to have CB_1_/CB_2_ antagonist properties and appears to decrease the THC-CB_1_ agonist activity in vitro [95]. However, the precise mechanism of the efficacy of CBD signaling is not fully understood yet. Similarly, CB_1_ antagonist effects of Δ^9^-THCV were observed, while high concentration appeared to be agonistic in a model of antinociception [93,96]. Other data showed that Δ^9^-THCV exerts antiepileptic and anticonvulsant activities, suggesting a CB_1_-mediated effect [97]. CB_1_ and CB_2_ represent important but not exclusive agonistic targets for the compounds Δ^8^-THC and THC, whereas CB_1_ plays the major role for psychoactivity [98]. There have been many controversial studies about the effects of THC on seizure activity: some studies have shown anticonvulsant properties of THC in maximal electroshock rat model, while in other experiments the opposite effect of THC was observed, initiating seizures in Fischer rats and B6C3F1 mice [99,100]. It was also reported that both Δ^8^-THC and THC significantly reduced the incidence of seizures on the first and second day of a 7-day administration in cobalt-epileptic rats [101]. CBN is another cannabinoid, which has shown its effectiveness to reduce seizures in a mouse model of maximal electroshock [102]. As a full agonist for both cannabinoid receptors, as well as inverse agonist for CB_2_ (depending on the concentration), it has though not been proven if the anticonvulsant effects of CBN depend on one of the cannabinoid receptors or rely on other targeting [98,103]. Both phytocannabinoids (CBG and CBC) are partial CB_1_/CB_2_ agonists, but with no anticonvulsant effects [104,105].

#### 4.1.2. G Protein-Coupled Receptor 55 (GPR55)

The orphan receptor GPR55, which was identified in 1999, is expressed in regions of the CNS, including the caudate-putamen, and peripheral tissue such as the intestines, spleen and adrenals. This receptor is also located in the hippocampus, in particular the dentate gyrus excitatory neurons where it is suggested to be a regulator of spatial learning and memory, and synaptic plasticity. The rat GPR55 is composed of 319 amino acids that share an amino acid identity of 67% with the human GPR55 [106,107]. This putative cannabinoid receptor is involved in anti-inflammatory effects in microglial cells, and proliferation of pancreatic cells and tumor growth in mice [108,109]. GPR55 activates intracellular Ca^2+^ release in neurons, which can alter neuronal excitability by stimulating glutamate release [110]. For this reason, antagonist activity could result in the shift of excitatory and inhibitory balance. Notably, CBD was shown to increase inhibitory transmission by blocking GPR55, which leads to an attenuation of epileptic seizures as seen in a mouse model of DS, a severe form of childhood epilepsy [111]. GPR55 antagonism has been evaluated in several studies as a potential treatment for refractory epilepsy. Besides, it has been observed that THC, Δ^9^-THCV, CBD, CBDV and CBG are able to block the response generated by the main endogenous GPR55 ligand lysophosphatidylinositol (LPI) and endocannabinoids [110,112]. However, THC and Δ^9^-THCV, which are partial and weak GPR55 agonists, might act through different targets and, for this reason, they could involve a variety of mechanisms.

#### 4.1.3. G Protein-Coupled Receptor 18 (GPR18)

Discovered in 1997, GPR18 is, as GPR55, described as putative and orphan cannabinoid receptor and was shortly considered to be named CB_X_ and CB_3_ receptor, respectively. Due to some missing criteria of the Nomenclature Committee of the International Union of Basic and Clinical Pharmacology (IUPHAR), these receptors were officially declared as orphan cannabinoid receptors [113,114]. In the mouse, GPR18 is located in a broad range of tissues including the cardiovascular system and the gastrointestinal tract, where it is involved in obesity/diabetes-associated inflammation and lymphoid system regulation. In humans, this receptor has not been as well studied as in rodents, but shows expression in the brainstem, hypothalamus, testis, spleen and lymph nodes [114,115,116,117]. GPR18 is composed of 331 amino acids and overlaps in amino acid identity with a similarity of 86% (mouse) and 85% (rat) to humans [118]. The downstream signaling of GPR18 is activated by *N*-arachidonoyl glycine (NAGly), which is a mixed agonist/antagonist endogenous ligand shown to activate intracellular Ca^2+^ mobilization [119]. The receptor is found on the cell surface of macrophages and microglial cells, as well as intracellularly, so to modulate the downstream signaling [120,121,122]. There is evidence that it plays a vital role in apoptosis of inflammatory leukocytes and is engaged in the reduction of intraocular pressure in mice [123,124]. As a GPR18 antagonist, CBD inhibits NAGly and was suggested to lower the effects of excitation to restrain the action potential firing. Abnormal cannabidiol (Abn-CBD) is a synthetic regioisomer of CBD, since it has the same functional group on a different position. It is a selective ligand and agonist for GPR18 able to induce a reduction in calcium release [125]. The selective and concentration-dependent ligand THC is able to induce proconvulsant effects by activating the calcium mobilization and, therefore, excitation by activating GPR18. Moreover, CBD was able to block the effects of THC in a simultaneous treatment. The evidence for the ability of CBD and THC to regulate the activity of GPR18 is still poor and does not allow a clear interpretation; however, there is a strong interest for the possible therapeutic use of these molecules [120].

#### 4.1.4. Opioid Receptor µ and δ

Opioid receptors are membrane receptors located in multiple regions of the CNS, including various hypothalamic nuclei, amygdala, hippocampus, substantia nigra, dorsal root ganglia, spinal cord, etc.; they are also peripherally found, as in the gastrointestinal apparatus [126]. Due to their broad range of involvement in numerous neurological modulations, such as mood disorders, pain perception and drug abuse, opioid receptors are widely explored [127,128,129]. THC and CBD might act as allosteric modulators of the opioid receptor subtypes µ and δ [130]. Antagonists of selective δ receptors were shown to diminish *N*-methyl-D-aspartate (NMDA) receptor-mediated seizures in vivo [131]. CBD revealed in multiple studies beneficial effects on massive uncontrolled glutamatergic firing, especially mediated by NMDA receptors. A study in 2018 proposed that CBD may act either by an unknown mechanism or as antagonist-like agent towards δ receptors to reduce NMDA receptor-induced seizures in vivo [132].

### 4.2. Transient Receptor Potential Vanilloid

In 2001, phytocannabinoids were first observed to interact and modulate the transient receptor potential vanilloid (TRPV) type 1 and 2 [133]. TRPV represents a subtype of the transient receptor potential channel (TRP), consisting of six transmembrane helices, a cation-permeable pore with intracellular N- and C-termini, allowing a calcium influx into the cell. TRPV1 (capsaicin-sensitive) and TRPV2 (capsaicin-insensitive) are widely located on distinct dorsal root ganglia neurons, trigeminal ganglia, peripheral afferent fibers and especially on nociceptive sensory endings, where they transduce pain, temperature, proinflammatory stimuli, and can be also activated by chemical substances, such as anandamide, vanilloids and cannabinoids [134]. As TRPV1 agonists, CBD rapidly dephosphorylates and desensitizes TRPV1 channels leading to a decrease in calcium influx and therefore reduced neurotransmission. There is evidence that TRVP1 channels are overexpressed in models of TLE and patients suffering from epilepsy [135]. Consistent with these observations, CBD presented reduced anticonvulsant properties in TRPV1 knock-out mice [136]. Patch-clamp analyses performed in HEK293 cells revealed that CBD and CBDV activated and desensitized TRPV1 and TRPV2 in a dose-dependent manner. In addition, CBDV was able to significantly decrease the amplitude and duration of epileptiform neuronal spikes [137]. In a model for juvenile seizures, CBDV was able to suppress seizures induced by pentylenetetrazole at postnatal day 10 (P10) in rats. Otherwise, in P20 rats CBDV decreased seizures induced by pentylenetetrazole or methyl-6,7-dimethoxyl-4-ethyl-β-carboline-3-carboxylate administration, and also by maximal electroshock stimulation, in agreement with the results obtained in P20 TRPV1 knockout mice with the same drug. These findings show that the effects of CBDV in different ages and epilepsy models are TRPV1-dependent [138]. Other phytocannabinoids including CBN, CBG, CBC and Δ^9^-THCV show TRPV type 1-4 agonistic activity, but a correlation to epileptic behavior has not yet been found [139,140]. These results will lead to further basic research on targeting TRPV1 and TRPV2 to test their promising potential in clinical treatment of epilepsy.

### 4.3. GABA_A_ Receptors

The GABA_A_R was first detected in the brain in 1950 and is a member of the pentameric ligand-gated ion channels superfamily [141]. To date, there are 19 different subunits present in the mammalian brain: six α (α1–6), three β (β1–3), three γ (γ1–3), and δ, ϵ, θ, π and ρ_1_ to ρ_3_, which form the heteromeric GABA_A_ receptors providing a wide spectrum of pharmacological and physiological characteristics [142]. When GABA, which mediates the main inhibitory neurotransmission in the brain and spinal cord, binds to GABA_A_R, the rapid influx of Cl^−^ is activated resulting in hyperpolarization and inhibition of the cell [143,144]. Those currents can be regulated by several positive and negative allosteric modulators such as benzodiazepine, neurosteroids, zinc and phytocannabinoids. The composition of the subunits of GABA_A_R is responsible for the sensitivity to those modulators, meaning that a shift in subunit composition could alter the functioning of GABA_A_R and lead to unexpected neuronal impairment [145]. GABA_A_ receptors are expressed on synaptic and extrasynaptic sites of the most, if not all, neurons in the CNS and were previously found to be also located on human peripheral blood mononuclear cells (PBMC) [146]. They are considered as being the most crucial receptors for pharmacological and physiological alteration and contribute to the etiology of numerous neurological and mental diseases such as epilepsy, schizophrenia and Angelman’s syndrome [147,148,149]. Multiple studies proved that a change of GABA_A_R subunit composition occurred in a model of kainic acid induced TLE, as well as in hippocampal tissues from patients affected with TLE [147,150,151]. At a concentration of 100 µM, CBD acts as a positive allosteric modulator of GABA_A_R as showed by voltage clamp electrophysiological measurements in Xenopus oocytes. Interestingly, coadministration of CBD and clobazam resulted in a greater anticonvulsant potency than clobazam only, because it significantly enhanced GABA_A_R-mediated transmission in a mouse model of DS [152,153]. An additional study examined patients with DS who donated their brain tissue to be transplanted in Xenopus oocytes to prove that a low dose of CBD was able to significantly enhance GABA_A_ currents [154]. CBDV is yet an unknown GABA_A_R agonist but demonstrated in a similar preclinical study for TLE its anticonvulsant properties mediated by GABA_A_R. TLE tissue with hippocampal sclerosis was derived from pharmaco-resistant TLE patients and showed, after a prolonged incubation with CBDV, a recovery of the current rundown of GABA_A_R [155]. However, there is still little evidence about the anticonvulsant effects of CBDV mediated by GABA_A_R.

### 4.4. Voltage-Gated Sodium Channel (VGSC)

VGSC (Na_V_s) were discovered by Hodgkin and Huxley in 1952, when examining the excitation and conductance in axons of giant squids and were first isolated from the eel electroplax [156,157]. They are members of the cation channel superfamily and are responsible for the Na^+^ conduction through the cell plasma membrane. Mammalian VGSC are composed of a large pore-forming α-unit that associates with one or two β subunits and have been found in almost every type of neuron examined. VGSC subunit α has nine known subtypes, which show tissue specific expression properties. The subtypes Na_v_1.1, Na_v_1.2 and Na_v_1.3 are primarily located in the CNS and peripheral neurons, whereas the other members are expressed in skeletal, cardiac muscles and interstitial cells of Cajal [158]. Mutation in the gene SNC1A, which encodes Na_v_1.1 results in cognitive impairment and causes due to the deficient sodium channel shift in neuronal excitability, resulting in phenotypes known as generalized epilepsy with febrile seizures. Furthermore, this mutation—occasionally in combination with GABA_A_R impairment—is carried by the majority (70–80%) of patients with DS [159,160]. Further mutations in Na_v_1.1, Na_v_1.2, Na_v_1.3 and Na_v_1.6 have been linked to the occurrence of epileptic seizure in patients of all ages [161,162,163]. Acting as an agonist, CBD appeared to inhibit and block the opening of Na_v_1.1 to Na_v_1.7 with low µM potencies, measured in human cell culture and rat brain slices [104]. In a Hodgkin-Huxley model of cortical neuron, CBD could decrease and stabilize neuronal excitability [164]. A different study showed that CBD was able to preferentially target and inhibit aberrant and increased resurgent currents in mutations in Na_v_1.6. Moreover, CBD demonstrated to diminish overall action potential firing of murine striatal neurons, assuming the high potential of treating drug-resistant children affected from DS who carry gene mutations in VGSC with CBD [165].

## 5. Isolated Phytocannabinoid versus Cannabis Extract: the “Entourage” Effect

The application of individual phytocannabinoids isolated from cannabis extract in therapy has recently sparked widespread debate [166], in spite that Mechoulam elucidated the structure of THC in 1964 and a drug based on the stereoisomer produced by cannabis, THC (Dronabinol), was approved by the FDA as safe and effective drug for HIV/AIDS-induced anorexia and chemotherapy-induced nausea and vomiting [167]. This is because the employment of cannabis or its medicinal extracts is still widespread, since anecdotal evidence indicates that cannabis extracts are more potent and with less side effects than THC itself. Several recent works indicate that other components of the cannabis extract may somehow interact with THC [166,168,169,170,171]. Studies in humans and animals suggest high potential for CBD to attenuate the effects of THC, in particular in decreasing the effects of THC on cognition/memory [172,173,174]. Conversely, there are preclinical studies that indicate that CBD may potentiate some effects of THC [165,175,176,177,178,179,180]. However, the few studies about the CBD-THC interaction represent a profound lack of research respecting the manner how CBD may affect behavioral and physiological effects of THC.

As THC, CBD was also formulated as a single active substance drug [181]. But also, in this case the use of hemp (cannabis for fiber of chemotype III) extract is widely considered for both nutraceutical and medicinal purposes. A recent study proposes that hemp extract requires a dose of CBD four times less than the drug containing the single CBD molecule to achieve the same therapeutic effect [182]. In this article it is clearly indicated how the use of hemp extract leads to a lower incidence of the adverse effects observed when administering the purified CBD. E. Russo refers to this synergy as an “entourage” effect [166]. The main problem with the use of hemp extracts is the poor knowledge of the entire chemical composition. Over 500 compounds and over 200 terpenes have been identified in cannabis so far [183]. Each of these compounds could be present in the hemp extract and could influence the pharmacological activity of CBD. Furthermore, the non-compliance of strict rules of drug preparation, could lead to high concentrations of THC in the extract with side effects typical of the latter compound.

It is challenging to identify the absolute chemical composition of the hemp extract even if, thanks to recent sophisticated analytical techniques, it is possible to determine a large quantity of substances present even at very low concentrations.

In conclusion, the use of CBD extract certainly offers advantages over isolated CBD, but it becomes difficult, if not impossible, to standardize it for each of its chemical components with consequent variability in pharmacological action.

## 6. Conclusions

For thousands of years the cannabis plant has represented a significant medical and economic value and is used all over the world. With the first preclinical experiments solely using selected compounds widened the enormous impact on alternative treatments for neurological diseases. *Cannabis sativa L*. stepped in the main focus of present research and reached an approximately 2400-fold publication rate since the first official cannabis report in 1939. While the first experimental approaches of THC and CBD have revealed possible beneficial health effects in general, previous studies focus more on distinct targets as well as signaling cascades in diverse models of specific diseases. Therefore, more and more derivatives of the plant were isolated and tested on a molecular and behavioral level, leading to the knowledge that most phytocannabinoids act through a broad spectrum of targets, which complicates the understanding of their exact pathway and action. THC and CBD have shown in numerous preclinical studies to diminish duration, severity and incidence of epileptic seizures in combination with remote adverse effects. With the approval of the first cannabis-derived medical drug in 2018, Epidiolex^®^ has fought its way from the laboratory to patients of all ages suffering from refractory epilepsy depicting a high success rate. The severe forms of pharmaco-resistant childhood epilepsy, DS and LGS respond to Epidiolex^®^ with a 36–50% amelioration rate; a milestone in epilepsy research [68,184]. Even though the anticonvulsant efficacy of different phytocannabinoids including CBD, THC, Δ^8^-THC, Δ^9^-THCB, Δ^9^-THCV, CBDV, CBN have been proven, the precise mechanism and modulation of targets depicted in this review opens many questions. Most phytocannabinoids are not only restricted to the modulation of a single receptor, but rather have the ability to regulate various receptors and channels and might therefore change the entire circuitry. In the past years, researchers have identified further targets of cannabinoids such as serotonin receptor (5HT_1A_, 5HT_2A_, 5HT_3A_), GPR12, glycine receptor, acetylcholine receptor, peroxisome proliferator-activated receptors, α_2_ adrenergic receptor, equilibrative nucleoside transporter and VGCCs. Until now there is no evidence that cannabinoids have beneficial effects on epilepsy acting through those receptors or channels and still remain to be examined.

Recently, two new compounds have been first isolated from the cannabis plant: the butyl and heptyl homologs of THC: Δ^9^-THCB and Δ^9^-THCP, respectively. Regarding their characteristics showing high CB_1_ affinity and overall high cannabimimetic activity, those new derivatives represent promising tools in the research of neurological diseases and especially epilepsy, which needs to be established in future studies [50,185].
